# A Systematic, Knowledge Space-Based Proposal on Quality by Design-Driven Polymeric Micelle Development

**DOI:** 10.3390/pharmaceutics13050702

**Published:** 2021-05-12

**Authors:** Bence Sipos, Gábor Katona, Ildikó Csóka

**Affiliations:** Faculty of Pharmacy, Institute of Pharmaceutical Technology and Regulatory Affairs, University of Szeged, Eötvös Str. 6, H-6720 Szeged, Hungary; sipos.bence@szte.hu (B.S.); katona.gabor@szte.hu (G.K.)

**Keywords:** Quality by Design, polymeric micelle, risk assessment, quality management, knowledge phase, preformulation space

## Abstract

Nanoparticle research and development for pharmaceuticals is a challenging task in the era of personalized medicine. Specialized and increased patient expectations and requirements for proper therapy adherence, as well as sustainable environment safety and toxicology topics raise the necessity of well designed, advanced and smart drug delivery systems on the market. These stakeholder expectations and social responsibility of pharma sector open the space and call new methods on the floor for new strategic development tools, like Quality by Design (QbD) thinking. The extended model, namely the R&D QbD proved to be useful in case of complex and/or high risk/expectations containing or aiming developments. This is the case when we formulate polymeric micelles as promising nanotherapeutics; the risk assessment and knowledge-based quality targeted QbD approach provides a promising tool to support the development process. Based on risk assessment, many factors pose great risk in the manufacturing process and affect the quality, efficacy and safety profile. The quality-driven strategic development pathway, based on deep prior knowledge and an involving iterative risk estimation and management phases has proven to be an adequate tool, being able to handle their sensitive stability issues and make them efficient therapeutic aids in case of several diseases.

## 1. Introduction

Research and development (R&D) has changed enormously in recent decades and leaders of pharma companies are in the midst of unprecedented change. With the increased prevalence of chronic pulmonary diseases due to pollution, vascular and neurological diseases from obesity and the aging population pyramid and, last but not least, the occurrence of pandemics such as COVID-19 or Ebola accelerate pharmaceutical manufacturing and research processes that must be adhered to strict quality conditions. A newly manufactured and market-placed medicine has to face obstacles for the industry players. Changes in consumer attitudes, cybersecurity threats, rapid advances in technology, non-adequate return on innovation and competition from companies in emerging economies are several of them [1,2]. In the 21st century, a novel quality assurance approach, Quality by Design (QbD)), appeared, oriented towards quality of the product and the improved patient applicability during therapeutic use. Quality, safety and efficacy are the three cornerstones of the QbD approach, which is built around starting from preformulation studies all the way to the patient’s own use [3,4].

With the development of nano drug delivery systems (nanoDDS), the previous quality assurance systems were no longer sufficient, which also supports the QbD method based on quality, knowledge and risk assessment [5]. Lipid-, polymer-based or other nanoDDSs all require a specific regulatory environment [6,7]. On the one hand, the reason for this is to be found in the production process, as conventional production line equipment is not necessarily suitable for the production of these products besides the need of special conditions and constant control. The opportunity is there in the hands of all developers to apply this method and in the course of our research group, the existence was proven on many occasions for nanoDDSs, such as liposomes [8], polymeric micelles [9] and human serum albumin nanoparticles [10]. On the other hand, safety must also be proven, as an increase in efficacy is expected due to the advanced properties of the systems, but in the case of improperly optimized preparations, it can lead to nanotoxicity and severe side effects [11,12].

Polymeric micelles are self-assembling association colloidal systems composed of amphiphilic copolymers capable of entrapping hydrophobic drugs [13]. They have a number of advantages, perhaps the most important of which is the increase in water solubility, along with a reduction in particle size. Together, these two factors contribute to a more favourable pharmacokinetic profile and to the incorporation of certain poorly soluble or penetrating active pharmaceutical ingredients (APIs) into a liquid dosage form [14]. The physical stability of the polymeric micelles proves to be adequate and they are able to maintain their particle size even during the circulation time in the bloodstream. In addition to administration by alternative administration routes, they show increased resistance to the body’s metabolic effects, which is also important for maintaining the active, effective form of the API [15]. For safety and quality assured development, every developer must consider all possible outcomes before, in between and after the preparation of nanotherapeutics, which needs a holistic and systematic knowledge space-based development [16]. As mentioned, the leaders of pharma industry must create innovative dosage forms in order to stand a chance in the global market. To achieve this, polymeric micelles are an excellent solution. Compared to liquid formulations, these nanosized systems have the higher ground when talking about decrease of dosage strength; therefore, the occurrence of side effects, the ability to offer higher permeability and flux values across biological membranes and, last but not least, the pleasing carrier integrity through the circulation in the body.

The aim of this research article is to collect and evaluate the critical parameters affecting polymeric micelles as promising drug delivery systems as part of the extension of QbD to the early development phase of pharmaceutical R&D processes [17]. This article develops the formulation space through the adaptation of literature date into exact quality-influencing factors. Based on our previous experience from formulation studies in the field of nanotechnology and polymeric micelles supplemented by literature data, quantified risk severities based on this systematic summary was calculated in order to help and encourage professionals in the pharmaceutical field to apply QbD in the development processes. Later, described possible Quality Target Product Profile (QTPP) and Critical Quality Attributes (CQAs) were collected and an extensive risk assessment (RA) was performed on them. The Critical Process Parameters (CPPs) and Critical Material Attributes (CMAs) went through a RA also and because many formulation methods exist in the development of polymeric micelles, these methods were evaluated individually.

## 2. Materials and Methods

### 2.1. General Methodology of QbD

**Figure 1:**
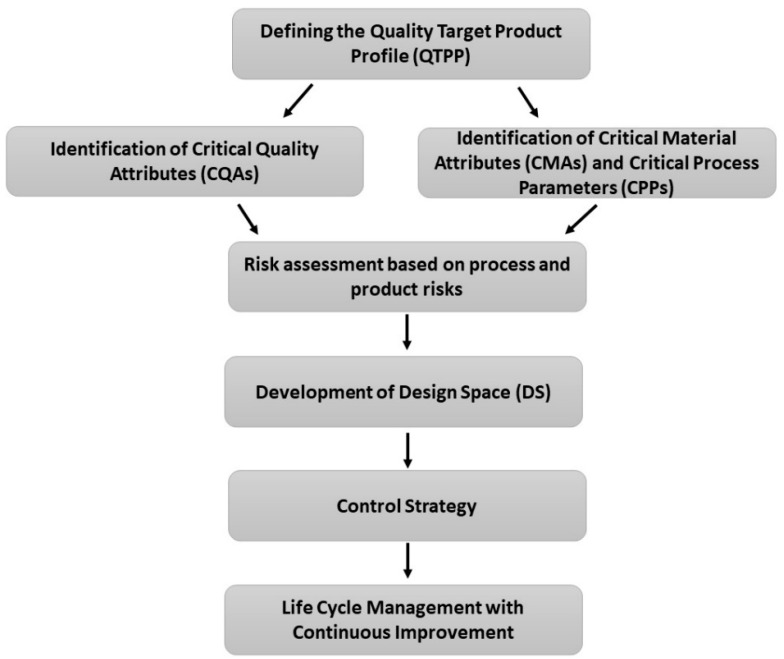
Schematics for the Quality by Design methodology.

Based on specified guidelines of the International Council of Harmonisation of Technical Requirements for Pharmaceuticals for Human Use [18,19,20], the QbD procedure followed the following steps (Figure 1).

At first, QTPPs were determined representing the desired quality characteristics of a finalized API-loaded polymeric micelle. The ICH guidelines state the mandatory QTPP elements such as indication, route of administration etc. and other safety and efficacy influencing factors were taken into account [18]. CQAs, CPPs and CMAs were selected in the next step. CQAs are physicochemical, biological or microbiological characteristics which should be controlled in an appropriate range to ensure product quality. CPPs and CMAs are related to the production of the product, which should be monitored [18,19,20]. Based on the collection process, a risk assessment was evaluated [20]. Data from the literature were collected using journal publishers’ search engines.

### 2.2. Risk Assessment Procedure

As QbD is a knowledge- and risk assessment-focused approach, qualitative and quantitative risk factors, expressed as severity scores, must be presented [17]. LeanQbD Software (QbD Works LLC, Fremont, CA, USA) was used for the RA procedure. At first, an interdependence rating amongst the QTPPs and the CQAs and the CQAs and the CPPs was performed. A three-level scale was used to describe the relation between these parameters: each relation was assigned with a “high” (H), “medium” (M) or “low” (L) attributive. The decision of assignment was performed based on numerous aspects including: occurrence of the risk factor during formulation and/or final product development phase; controllability of the factor; whether the factor can be eliminated or can it be fixed at a certain value without affecting quality; detectability. The description of relations had the fundamental basis of how closely the factors are related and data from the collected literature, in addition to the regulatory aspects of a joint MHLW/EMA reflection paper on the development of block copolymer micelle medicinal products [21]. Using the software, these qualitative relations were the basis of calculating the severity scores. As the output of the RA, Pareto diagrams were generated, presenting numeric data and ranking of the CQAs and the CPPs representing the potential impact on the final product [22].

## 3. Results

### 3.1. Collection of QTPPs for Polymeric Micelle Development

The definition of QTPPs is the first major step in the development of nanoDDS; in this case, polymeric micelles. In addition to the mandatory elements of the ICH guidelines, optional QTPPs can be defined, which mainly depends on the nature of the target product. Defining the indication is of paramount importance. This QTPP is most often due to the properties and previous application of the API. By forming a polymeric micelle formulation, the indications in the previous SPCs can be also supplemented [23]. Achieving this plays an important role mainly in immune and cancer therapy [24]. On the other hand, by innovating the mode of application, the API can be delivered to biological spaces which previous conventional formulations are not capable of. A particularly good example of this is the delivery of non-steroidal anti-inflammatory drugs to the central nervous system (CNS) for the treatment of neuroinflammation, in which case, conventional peroral formulations do not or only a small extent can enter [25].

The patient population as QTPP is also a mandatory clarifying factor that can be expand beyond the age associated with the general drug. The development into a liquid dosage form is beneficial for the patient, as the product can be used even in case of swallowing difficulty. This is especially important in pediatric and geriatric medication, where patient experience is of paramount importance to improve therapy fidelity, the so-called adherence [26]. Polymeric micelle formulations designed for a conventional route of administration are aimed primarily to increase the bioavailability of products previously authorized for that route. However, new development trends prefer alternative routes of administration, of which the ophthalmic, topical and nasal delivery play the largest role [27,28,29]. A significant number of active substances are able to exert their effect only at certain point of action in the body, in the given indication. However, this does not mean that their potential is not greater and nanoformulations are able to deliver APIs to new sites. Of these, the intake of the drug into the CNS and cancer cells should be highlighted [30,31]. The dosage strength of the formulation depends on many things: indication, API features and the administration route. Manufacturers should clearly indicate the proposed dosage form of the formulation because they have different stability and product quality in colloidal solution, colloidal gelling system or freeze-dried form [14].

Physicochemical characteristics are common amongst QTPPs as well, depending on the indication and the administration route. Most commonly, viscosity, osmolality, pH and mucoadhesive properties are presented. The particle characteristics of polymeric micelles include the particle size (expressed as Z-average), particle size distribution (expressed as polydispersity index, PdI), zeta potential, encapsulation efficiency (EE%) and drug loading (DL%). These are the factors that facilitate the implementation of the other QTPP elements. They can also appear as CQAs, but in the scope of regulation of nanosystems, these factors must be defined in the initial plan, as part of the basis of technological innovation lies in their regulation [8,12].

The properties of the copolymers and the API determine the safety profile of the target product the most. The main criterion for polymers is that they are biocompatible with the human body and that, after their metabolism, the degradation products do not cause harmful side effects [32]. Where necessary, sterility is also required, as well as the product characteristics that allow the safe use of the dosage form and the administration route. The stability of polymeric micelles can be approached in several ways. Stability in aqueous solutions means a constant particle size after redispersion and a reduction in the tendency to aggregate. In addition to the liquid form, it is also worth checking these parameters during storage after freeze-drying [14]. Some copolymers are temperature and pH sensitive, so it is important to know whether a particular product can provide the expected effect under these factors. Changes in structure are expected after drug loading and which are also critical parameters of product characteristics. The classification of solubility among QTPPs is also a common case, as reducing redispersion time, improving solubility rate and increasing wetting properties are also the goal in the production of polymeric micelles. This is closely related to drug release, such as QTPP, where different aims can be achieved. Most commonly, the aim is the rapid onset of action, but in other cases, sustained release with prolonged circulation time can be accomplished after modifying the polymeric micelle structure [33,34]. Drug release is also influenced based on indication, the route of administration and the API itself. Drug permeability is included as QTPP, aiming either to enhance passive diffusion or the carrier-mediated active transport mechanisms [35]. The described QTPPs are summarized in Table 1.

### 3.2. How to Choose the Proper QTPP Elements for the Research Project?

When QbD comes to the fore as a tool to use in research, the first step is to gather the QTPP elements; however, the question arises as to which ones are needed within the general elements or whether another one could be added to the QbD setup. Many questions must be answered in the beginning which will help the determination of QTPPs:

- With the formulation, do I change more than the physicochemical properties of the API?
- What would the final dosage form? What are the requirements for it?
- Is there something similar in the literature or on the market? What more can I add to the formulation?
- Who should be the recipients of my formulation? What are the criteria for that target population?
- Do I possess all the necessary tools in my experiments to validate my claims and to characterize the nanocarrier?
- What is the chosen dosage strength and what pharmacokinetic changes can be expected? How do these changes affect the target population and do I need to make adjustments?
- What can the polymers, surfactants and other excipients do? What are their main physicochemical and colloidal properties and how can I utilize them?
- What are the possible toxicological aspects of the formulation? Is the drug content or polymer concentration below a safe level?
- To what extent can I optimize the formulation and can I perform a scaling up?
- What are the drug product quality criteria (e.g., purity, sterility) to be used in clinical settings or to place them on market?
- What container closure system will be used and can the formulation adapt to that?
- What else can the formulation be used for besides my target?

The list of questions could go further based on the research project itself, but quality management tools such as an establishment of an Ishikawa diagram or mind map are encouraged to be utilized for selecting the QTPP elements [36].

### 3.3. Regulatory Reflections

The European Medicines Agency (EMA) published a joint MHLW/EMA reflection paper on the development of block copolymer micelle medicinal products, which contains aspects to take into consideration when applying QbD as well [21]. It is supplemented by the general ICH specifications, e.g., the guidelines of safety pharmacology studies for human pharmaceuticals (ICH S7A) [37] and the general development and manufacture of drug substances (ICH Q11) [38]. There are multiple factors that should be taken into account: first, the description and composition which are related to the later described CQA and CMA elements. Such factors are the content of the block copolymer and the active substance in the product, the composition and the polymer’s material attributes, followed by the quality characterization of the product. Typical examples are the impurity profile, the micelle size, morphology, surface properties, drug loading and in vitro release/permeability profile, which can be generally measured to all polymeric micelle product. The joint paper also states that the stability of the block copolymer micelle products should be based on the ICH Q1A(R2) [39] guideline and for the ones containing biological or biotechnological entities, the ICH Q5C can be applied [40]. For example, if the stability is included in the QTPP profile of the product, it should be more specific depending on the manufacturer’s goal—whether it should be physically and/or chemically stable—which is more important or, in other words, based on the material properties of the building agents which have the higher risk value. Non-clinical pharmacokinetics should include relevant measurements of the release and permeability profile of the product complemented by thorough active substance and micelle-forming polymer metabolite screening using biorelevant mediums (maybe organs and/or tissues). When applicable, safety pharmacology studies should be conducted in accordance with ICH M3(R2), ICH S7A and ICH S7B [37,41,42] with toxicological studies based on ICH S4, ICH S6(R1) and ICH S9 [43,44,45]. The ICH M3(R2) guidelines also define the starting dose for first-in-human studies. By keeping in mind this short summary and the knowledge of the specific guidelines, the CQA and CMA elements were collected in the next step.

### 3.4. Collection of CQAs for Polymeric Micelle Development

CQAs include those elements that define the quality of our main objectives, the physical, chemical or microbiological characteristics required to achieve the optimal formulation. During screening processes, two main things need to be determined or examined first: the nature of the polymeric micelle forming copolymer and the type of polymeric micelle. Copolymers can differ in the constitution of monomers and the sensitivity towards environmental changes. A distinction can be made between copolymers consisting of two block and those consisting of three blocks, the latter of which can be composed of two or three different monomers. Graft copolymers are considered to contain chain branches allowing a more diverse structure [46]. Innovative copolymers are already able to respond to stimuli by altering the tertiary or quaternary structure of the polymer. Such a stimulus can be, for example, pH, temperature, ionic strength or even binding to an immunological element [47]. The arrangement of conventional polymeric micelles is as follows: in a polar liquid medium, the hydrophilic side chain is solubilized to the outside world, while the hydrophobic moiety keeps the drug molecularly dispersed. The inverse of this can be also distinguished when talking about a reverse micelle. Mixed micelles are polymeric micelles in the construction of which several copolymers play a role. The polymer side chains can take various forms, depending on the material properties, such as a flower-like micelle, unimolecular star micelle or unimolecular dendritic micelle. In the hydrophobic core of a polymeric micelle, several compartments can be formed, mainly with oily substances, in which case, it is a multicompartment micelle [46]. Morphology of the polymeric micelles are influenced by the dosage form (e.g., they are incorporated into a hydrogel or just a colloidal solution) and whether surface modifications have been formulated on the surface of the outer shell. These modifications all have the same aim: to improve or control the pharmacokinetic profile of the drug release from the carrier [48,49].

Among the CQAs, the factors describing the particle characteristics of the polymeric micelles are included. Such factors are: particle size, polydispersity index, encapsulation efficiency (EE%), drug loading (DL%) and zeta potential. These, combined with polarity and the definition of wetting parameters, are import elements of optimization. The average particle size of polymeric micelles is between 20 and 200 nm, which is influenced by the properties of the building copolymers and the API itself. A particle size distribution is claimed to be optimal if it is below 0.300. Both the EE% and the DL% are strongly related to the material properties, the extent of which is determined by the quantitative and qualitative features of the polymer. As the hydrophilic side chains are oriented towards the outer surface after encapsulation of the API, the values of the contact angles to water will decrease, while those to hydrophobic materials will increase. This leads to increased polarity, which is closely related to water solubility and thus also to dissolution parameters [50]. These factors are the ones that most often form part of the design of experiment (DoE) as to be optimized. Optimization can be performed by factorial experimental designs, e.g., Box–Behnken design, central composite design, Plackett Burman design or the traditional 2^x^ DoE experiments. Nowadays, the creation of neural experimental design networks is also of key importance, as it is able to study the interactions between factors more broadly [51,52].

Further investigable CQAs include quantitative parameters related to the permeability of a carrier, for example flux, permeability coefficient (K_p_) or to the drug release, e.g., whether a specific kinetic model can be fitted to the dissolution curve or not. Sterility and stability are included as CQAs if they are of high significance, but the same variables are taken into account as they were QTPPs. The possible CQAs of polymeric micelles are summarized in Table 2. Choosing the proper CQA elements for the desired product depends on the later collected material attributes as well and they should not be considered equally potent and relevant to the development process; these differences should be highlighted and calculated later during an RA.

### 3.5. Collection of CPPs and CMAs for Polymeric Micelle Development

The precise definition of the CPP and CMA elements helps in the DoE process as these are the fundamental factors in getting from the raw material to the nanoDDS. In the case of polymeric micelles, the preparation can always start or end in a liquid medium, where four main factors are distinguished: the copolymer, the API, the solvent(s) and any excipients added. The general CMA factors are presented in Table 3.

Several methods have been developed for the production of polymeric micelles. Direct dissolution method is commonly used when the micelle-forming copolymers have relatively high water solubility. The API and the copolymer are directly dissolved in an aqueous media associated with stirring, heating and/or sonication in order to form the drug-loaded polymeric micelles [53]. Thus, micelle formation happens by the dehydration of the core forming blocks. The dialysis method is for copolymers with low water solubility; therefore, organic solvents are used in this method. During the preparation, the API and the copolymer(s) are dissolved in a common organic solvent and they are subsequently dialyzed against water. This triggers the micelle formation besides washing away residual organic solvents from the formed polymeric micelle solution [54]. Oily substances can be used in the preparation, when the API is miscible or soluble in the oil phase. With constant mixing, polymeric micelles can form in the aqueous phase of the emulsion, but this method needs many after cleaning processes [55]. The freeze-drying method is associated with dimethylacetamide or tert-butanol as organic (co-)solvents because of their high vapor pressure offering rapid sublimation. After dissolving the API in the organic solvent-water mixture, a freeze-dried cake of polymeric micelles can be achieved which demonstrates adequate shelf-life with high water dispersibility [56]. The thin film method is a production method based on complete solvent evaporation, where the drug and polymer system dissolved in any solvent (mixture) is recovered in the form of a thin film. It is then followed by hydration in water (or water-based buffer) and depending on the properties of the polymer, the process can also be followed by ultrasonication, mixing or even filtration, just like in the production process for liposomes [8,9]. Possible CPP elements and specific CMAs associated with each production method are collected in Table 4.

### 3.6. Risk Assessment on Critical Quality Attributes

**Figure 2:**
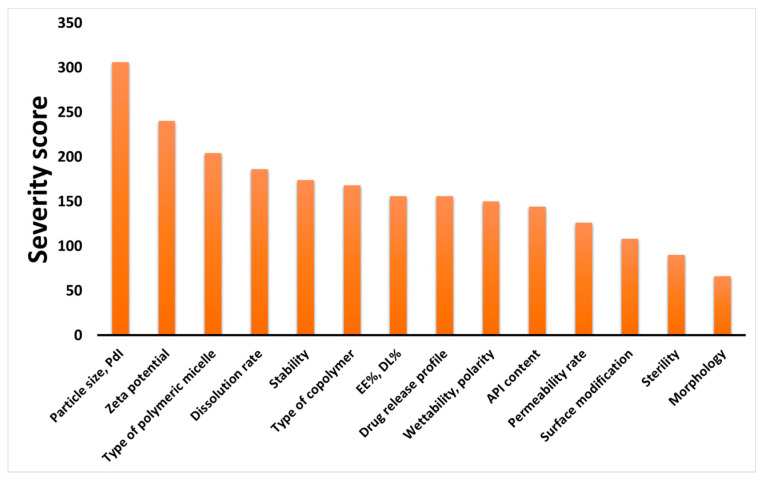
Pareto diagrams of the calculated severity scores of critical quality attributes (CQAs). Abbreviations: PdI, polydispersity index; EE%, encapsulation efficiency; DL%, drug loading; API, active pharmaceutical ingredient.

Based on the quality by design methodology, two risk assessment have to be performed: between QTPPs and CQAs, where the severity score of the CQAs is obtained, and between the CQAs and CPPs/CMAs, where the severity score of the latter can also be calculated. The severity scores of CQAs in declining order are presented on the Pareto diagram in Figure 2.

Based on the calculated severity scores of the CQA elements, it can be seen that the particle characteristics are the most important to take into account in the formulation of polymeric micelles. These dependent factors pose a great challenge to achieve, but it comes with many advantages. By decreasing the particle size with uniform particle size distribution expressed as polydispersity index (PdI), favorable solubility, wetting parameters, dissolution and permeability can be accomplished. Zeta potential is also of paramount importance, as the surface charge is directly connected to the physical stability in colloidal aqueous solution form.

### 3.7. Risk Assessment on Critical Process Parameters and Critical Material Attributes

**Figure 3:**
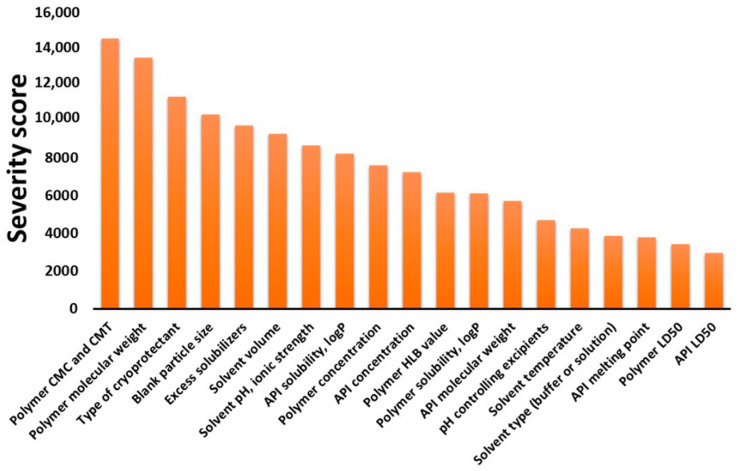
Pareto diagram of the calculated CMAs. Abbreviations: CMC, critical micelle concentration; CMT, critical micelle temperature; API, active pharmaceutical ingredient; HLB, hydrophilic-lipophilic balance; logP, partition coefficient; LD_50_, median lethal dose.

The second step of the risk assessment was divided into two parts. Firstly, the factors included as CMAs of the preparation processes were taken into account and, then, the CPPs/CMAs were examined, broken down into each preparation process. The Pareto diagram of the calculated severity scores of CMAs are presented on Figure 3.

**Figure 4:**
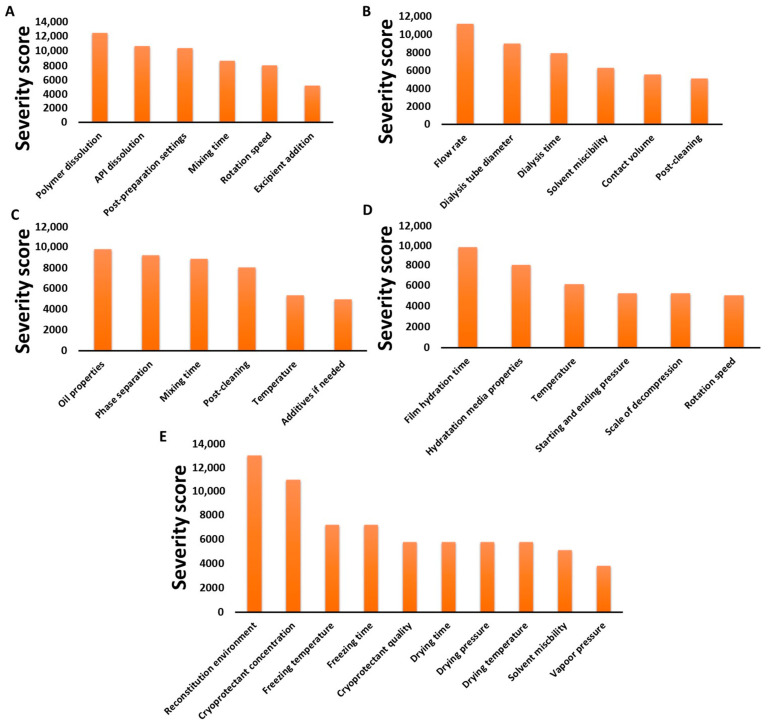
Pareto diagrams of the calculated CPP/CMAs: direct dissolution method (**A**), dialysis method (**B**), oil-in-water emulsion method (**C**), thin film hydration method (**D**) and freeze-drying method (**E**).

The material properties of the polymer, the API, the solvents and other excipients were taken into account in the risk assessment. The factors also cover data from the basic physicochemical property to the safety elements. Safety numbers are presented with low severity scores, as dosage strength is highly controllable, making it able to present a polymeric micelle formulation with less chance of toxicity. The polymer, API and solvent properties cannot be ever excluded, as they are severely influence the formation of the nanoparticles. On Figure 4, Pareto diagrams of CPPs/CMAs for each preparation method are presented.

It can be seen that quite different factors also appear on the different charts in decreasing order of severity score. Although the production methods may be based on a similar principle, it is important to assess the risks associated with each sub-step as part of the manufacturing process. Furthermore, the risk factors that may arise mainly from human negligence, e.g., measurement error, incorrect data setting or risks due to manufacturer/measuring instrument failure, e.g., broken device faulty circuit, are not presented as they are constant among the possible risk factors in the manufacturing process.

### 3.8. Case Example for the Thought Process of Applying QbD-Based RA

In the rapidly evolving field of nanotechnology and the manufacturing of polymer-based nanocarriers, the assigned severity scores can vary based on many material and target quality-based factors. It can be claimed that there is an antipsychotic compound which has low water solubility and inefficient permeability can be experienced from the intestinal tract from previously formulated solid tablet formulations. Based on this sentence alone, many QTPP elements can be already determined: What is the marketed/clinically efficient dosage strength of the API? What other administration route can I exploit? What is the target population of this API? What is the major indication field of this substance? What physicochemical and pharmacokinetic attributes do I want to improve on? The next step is to make adjustments to this based on your goal: instead of the adult population, the target population should be children; therefore, my target dosage strength must be lowered accordingly to pharmacokinetic parameters. A novel article was found during the knowledge space development phase of my research where the suspension form of this API delivered by the nasal route showed higher brain concentrations, resulting in more advanced therapeutic effect in the major indication. Then, all process and material parameters should be taken into account, for example, in the research laboratory, we have a deep knowledge on freeze-drying production method for polymeric nanocarriers, so that should be the chosen formulation method and there are suitable micelle-forming copolymers which are able to decrease the particle size of APIs below 100 nm.

Gathering all this information, we can now determine the QTPP profile of the desired product, meaning we want to develop a API-loaded mixed polymeric micelle, which:

- has the micellar size less than a 100 nm in monodisperse distribution (QTPP: particle characteristics);
- is suitable for the treatment of psychotic episodes (QTPP: indication);
- can be applied for children (QTPP: target population);
- is administered intranasally (QTPP: administration route);
- in the form of a nasal spray (QTPP: dosage form);
- in half of the currently marketed drug strength (QTPP: dosage strength);
- allowing higher brain concentrations (QTPP: site of activity),
- with sustained drug release (QTPP: drug release profile);
- and enhanced permeability across the nasal mucosa (QTPP: permeability profile).

After the collection of QTPPs, the next step is to gather all the possible CQAs relevant to these targets: mixed polymer types, particle size and polydispersity index, pH, viscosity, mucoadhesivity, osmolality, dissolution rate, permeability rate, drug content uniformity, etc., and investigate the effect on the QTPPs in a 3-scale interdependence rating followed by the calculation of the severity scores using any QbD-modelling software. The same applies to the CMAs and CPPs: for example, each mixed micelle forming polymer concentration, solvent volume, freeze-drying temperatures, cryoprotectant concentration and reconstitution. After the calculation of the severity scores, the following results were observed:

- The highest CQA severity scores were for the particle size, permeability rate and dissolution rate (based on the material attributes of the to be used excipients, polymers and the desired target of the product);
- The highest CMA/CPP severity scores were for the polymer ratios, the solvent volume and the cryoprotectant concentration (based on the establishment of knowledge about what does the equipment provided to us are able to perform and prior optimization processes for polymeric nanocarriers).

To continue next to set up a design of experiment, many aspects can be followed. One is to make one high-severity risk factor at a constant level or the other is to make it a dependent factor to our factorial design. We chose the cryoprotectant concentration to be fixed at 5 % *w*/*w* and, furthermore, we investigate the effect of the polymer ratios and the solvent volume on the independent factors: particle size, dissolution and permeability rate during a factorial design of our choosing.

### 3.9. Optimization Techniques of Polymeric Micelles via QbD-Based Design of Experiment (DoE)

One of the cornerstones of the QbD approach is that, at the end of the risk assessment, an extensive formulation optimization follows. This is most often done by setting up a factorial or neural experimental design, after which the design space is set up with proper statistical evaluation and then the formulation is characterized to validate it. If a quality defect is discovered during the life course of the formulation, then adjustments but be made. One good example of applying QbD as a part of the DoE set-up is the work of Khurana et al., where a 13-run central composite design was used to optimize resveratrol-loaded polymeric micelles [57]. Based on their risk assessment, the selected CQA elements with the highest risk severity were micellar incorporation efficiency (MIE), the particle size and the skin deposition potential. The effect of polymer ratio and the API content were investigated on these CQAs and a response analysis were carried out through polynomial equation. By plotting the surface plots, the optimized polymer to API ratio was found and satisfactory results were obtained: the observed MIE was 93.45% with a particle size of 142.67 nm and skin deposition of 51.63% which all meet the requirements of a polymer-based nanoDDS for topical drug delivery supplemented by advanced in vivo pharmacokinetics. Another great example of QbD-based DoE is the work of Lohan et al., where after the construction of an Ishikawa fish-bone diagram containing the factors influencing the galantamine-loaded mixed micelle formulation, a 7-factor 8-run Taguchi orthogonal array design was employed aiming to reveal the potential impact of various material attributes on product quality [58]. After the screening, they investigated the effect of DSPE-PEG 2000 and soy lecithin on the particle characteristics and drug release in a 13-run central composite design, as well as with 5 parallel measurements. A great tool to decrease the severity of the risk factors was applied by them: keeping the high-risk valued ethanol concentration at a fixed ratio, meaning that the optimized formulation should be matched with this factor. By the end of the optimization, the desirability functions were compared to the investigated parameters and a nanosized mixed micellar system was developed with a particle size of 175 nm, a zeta potential of −18 mV and a dissolution efficiency of 86%. In our previous work, the optimization of Meloxicam-loaded polymeric micelles via RA-based Box–Behnken factorial design was performed and a one-way ANOVA statistical analysis of the results was applied. The effect of the ethanol and sodium-hydroxide volume and the polymer concentration was investigated on particle size and particle size distribution with end results of 111.6 nm and a PdI of 0.114, allowing proper basis for nasal drug delivery [9]. In addition to these examples, many factorial/neural design studies are there in the literature for polymeric micelles; however, the lack of pre-optimization risk screening can be observed. That is why it is encouraging to use the demonstrated knowledge space development and risk assessment, along with statistical analysis of your choosing, so the micellar formulations can be matched to quality management as well.

## 4. Discussion

Through mandatory steps of quality by design, this article has collected and evaluated the factors that influence the successful development of a polymeric micelle based nanocarrier system. The QTPP elements, as proven, depend on the purpose; however, it is important to incorporate the mandatory elements of the ICH guidelines when setting up the pilot design. For polymeric micelles, as promising nanotherapeutics, this is not an easy task, as new indications and therapeutic possibilities may occur due to particle size reduction and modification of the administration route, leading to a different pharmacokinetic profile. The CQA, CPP and CMA elements were collected in their entirety to characterize a general formulation based on literature. These parameters are those that affect the quality of the product from the beginning of the manufacturing process to the patient’s use.

During the course of our research, we first collected the QTPP, CQA, CMA and CPP factors that influence the quality of a polymeric micelle formulation. Amongst QTPPs, it can be seen that it can range from general application principles to the extent of physicochemical and pharmacokinetic factors. It is important to see that QTPP elements are those without which risk assessment would not be possible. A separate risk assessment process is required for each polymeric micelle formulation, regardless of whether it contains an active substance or it concerns a development of a new polymeric carrier system able to form micelles. In the risk assessment with the CQA, CPP and CMA elements, we shed light on which posed the greatest risk for a general nanoformulation. Of course, a researcher has to decide for themself which factors are relevant to the production process and product quality chosen, so we performed the analysis for several production methods common amongst developers. The risk assessment of R&D processes is of particular importance for nanocarriers. Greater emphasis should also be placed on the basic properties of polymers and the active substance, as well as on the steps in the manufacturing process. The optimization of the particle characteristics also depends, crucially, on the nature of the building polymer and the manufacturing process.

Although the main points presented on the tables and figures are just fragments of the evidence presented in many articles and development processes, the systemic collection of all the influencing factors as data is the novelty of this current work. To demonstrate that the CMAs and CPPs parallelly improve the transparency between these parameters and their relation between CQA elements, in Table 1, Table 2, Table 3 and Table 4, the general collection of influencing factors can be seen, followed by an RA process as seen on Figure 2, Figure 3 and Figure 4. The Pareto charts on Figures show the theoretical ranking of the severity of the influencing factors as part of an initial general RA.

This presented work can fit well into the research area of polymeric micelles as it is an extension of the previous knowledge acquired by prior results. QbD is also recommended by regulatory authorities and encourage researchers to build the process of nanocarriers into their development.

## 5. Conclusions

The novelty of the work was that polymeric micelles were systematically evaluated on the basis of knowledge and risk, as these nanotherapeutics require a special regulatory environment. The quality by design methodology offered a thoroughly quality-focused assurance system, which proved to be useful in the case of polymeric micelles. As an encouragement, it is recommended that all researchers use the previous literature and evaluate the significant factors under a risk assessment process, resulting in a more focused design of experiment. Later, knowledge of these factors provides an important basis for quality and process control and allows for an easier to manage, correctable manufacturing process.

## Figures and Tables

**Table 1:** Systematic collection of feasible QTPP elements for the development of polymeric micelle nanoDDSs.

QTPP Element	Details
indication	general API-based extension of general API-based
patient population	paediatrics or geriatrics general, API-based
administration route	conventional e.g., peroral alternative e.g., nasal
site of activity	based on indication based on API new, feasible sites
dosage strength	based on severity of disease based on indication, API and administration route
dosage form	colloidal solution colloidal gelling system freeze-dried powder for further reconstitution
viscosity	based on indication and administration route
osmolality	based on indication and administration route
pH	based on indication and administration route
mucoadhesive properties	based on indication and administration route
particle characteristics	particle size (Z-average) particle size distribution (polydispersity index, PdI) zeta potential encapsulation efficiency (EE%) drug loading (DL%)
safety	biocompatibility degradation products dosage form related
stability	aqueous solution freeze-dried powder dilution stability pH and/or temperature stability structural stability
solubility	solubility rate wettability and polarity redispersion time
drug release	rapid onset of action sustained release prolonged circulation time based on indication, administration route and API
drug permeability	passive diffusion enhancement carrier-mediated active transport enhancement

**Table 2:** Systematic collection of feasible CQA elements for the development of polymeric micelle nanoDDSs.

CQA Element	Details
type of copolymer	diblock copolymer (A–B type)
	triblock copolymer (A–B–A type)
	triblock copolymer (A–B–C type)
	graft copolymer
	stimuli-sensitive copolymer
type of polymeric micelle	conventional micelle
	reverse micelle
	mixed micelle
	sensitive micelle
	flower-like micelle
	multicompartment micelle
	unimolecular star micelle
	unimolecular dentritic micelle
surface modifications	none
	polyethylene glycole (PEG) conjugates
	(monoclonal) antibodies
	peptids, lipids, carbohydrates
	pH or temperature sensitive sidechain
morphology	spherical
	star-like
	crew-cut
	semi-bald
particle characteristics	particle size, PdI
	zeta potential
	wettability, polarity
	EE%, DL%
API content	based on patient, administration route or indiciation
permeability rate	based on aim, described with flux or K_p_
dissolution rate	based on aim
drug release profile	kinetic or non-kinetic following
sterility	if needed
stability	same variables as in QTPPs

**Table 3:** Systematic collection of feasible CMA elements for the development of polymeric micelle nanoDDSs.

General CMA Element	Details
polymer properties	molecular weight
	HLB value
	critical micellar concentration and temperature
	concentration
	solubility, logP
	blank particle size
	LD_50_ value
API	molecular weight
	solubility, logP
	melting point
	concentration
	LD_50_ value
solvent medium	pH, ionic strength
	volume
	temperature
	buffer or solution
excipients	pH control
	excess solubilizers
	cryoprotectant if needed

**Table 4:** Systematic collection of feasible CPP elements for the different preparation methods of polymeric micelles.

Production Method	CPP/CMA Element
direct dissolution	API dissolution
	polymer dissolution
	mixing time
	rotation speed
	excipient addition
	post-preparation settings
dialysis method	flow rate
	dialysis tube diameter
	miscibility
	dialysis time
	contact volume
	post-cleaning
oil-in-water emulsion method	oil properties
	phase separation
	mixing time
	additives (e.g., other emulgents)
	temperature
	post-cleaning
freeze-drying method	cryoprotectant quality properties
	concentration of cryoprotectant
	solvent miscibility
	vapor pressure
	freezing temperature
	freezing time
	drying time
	drying pressure
	drying temperature
	reconstitution
thin film method/vacuum evaporation	temperature
	starting and ending pressure
	scale of decompression
	rotation speed
	duration
	film hydration time
	hydration media properties

## Data Availability

The data presented in this study are available on request from the corresponding author.
